# Early life cognitive function and health behaviours in late childhood: testing the neuroselection hypothesis

**DOI:** 10.1136/jech-2017-208896

**Published:** 2017-11-09

**Authors:** Daniel Aggio, Lee Smith, Mark Hamer

**Affiliations:** 1 Department of Primary Care and Population Health, University College London Medical School, London, UK; 2 The Cambridge Centre for Sport and Exercise Sciences, Anglia Ruskin University, Cambridge, UK; 3 School of Sport, Exercise and Health Sciences, Loughborough University, Loughborough, UK

**Keywords:** physical activity, cognition, smoking, alcohol drinking, child

## Abstract

**Background:**

Higher cognitive function in childhood is associated with healthier behaviours and a reduced risk of chronic disease in adulthood, but it is unclear whether this selection of healthier behaviours occurs in childhood or later in life. The present study investigated how cognitive function at age 3–7 years was associated with health behaviours at age 11.

**Methods:**

Verbal, non-verbal and spatial abilities were assessed using the British Ability Scales at ages 3–7. At age 11, children reported how often they engaged in sport/physical activity, sedentary behaviours (eg, reading and games console usage), cigarette smoking and alcohol consumption. Logistic regression was used to estimate odds of engaging in health behaviours at age 11 according to early life cognition.

**Results:**

A 1 SD increase in early childhood verbal ability was associated with reduced odds of attempting smoking in boys and girls (OR 0.69 (95% CI 0.57 to 0.84)) and reduced odds of computer gaming in girls (OR 0.79 (95% CI 0.72 to 0.86)) by age 11. Early childhood verbal ability was also associated with reduced odds of regular participation in sport/active games (boys: OR 0.91 (95% CI 0.84 to 0.99); girls: OR 0.81 (95% CI 0.74 to 0.88)) and increased odds of reading for enjoyment (boys: OR 1.47 (95% CI 1.35 to 1.60); girls: OR 1.48 (95% CI 1.36 to 1.62)) at age 11. Early childhood non-verbal ability was associated with reduced odds of alcohol consumption in boys and girls (OR 0.92 (95% CI 0.85 to 0.99)) and reduced odds of online messaging in boys (OR 0.89 (95% CI 0.81 to 0.98)) at age 11. Early childhood spatial ability was associated with reduced odds of participating in sport/active games in boys at age 11 (OR 0.88 (95% CI 0.82 to 0.95).

**Conclusion:**

Neuroselection may occur during early childhood resulting in some, but not all, healthier behaviours by age 11.

## Introduction

Cognition, or mental ability, in childhood has consistently been shown to be associated with a reduced risk of chronic disease^1^ and mortality^2^ in adulthood. One possible explanation for these observations is that individuals with higher cognition choose to engage in healthier behaviours, a theory known as neuroselection.^3^ Cognition in childhood has been associated with numerous health behaviours in adulthood. For example, higher mental ability at age 10 has been associated with a reduced risk of smoking,^4^ alcohol drinking problems,^5^ unhealthy dietary habits and physical inactivity decades later.^6^ Furthermore, executive functions such as inhibition, working memory and cognitive flexibility have been shown to be important for many aspects of life, including physical and mental health, quality of life and school and job success.^7^ One potential pathway that may explain these relationships is that individuals with higher cognition have an increased ability to appraise and act on health information.^8^

Current research in this field has focused only on outcomes in mid-adulthood.^1 3 5 6^ Thus, it is not possible to determine whether neuroselection occurs during or after childhood making it difficult to tease apart the effects of formal education. Understanding whether this phenomenon occurs during early life may help determine exactly when cognition is most important for the selection of health behaviours. If neuroselection occurs during early childhood, tests of cognition may help identify children who are most at risk of selecting into unhealthy behaviours earlier in life.

There is also evidence that some health behaviours, particularly physical activity, are neuroprotective, in that they are associated with higher levels of cognition years later.^9 10^ We previously showed that specific physical activities and sedentary behaviours are associated with higher levels of cognition later in childhood.^11^ However, it may be possible that some of these associations are explained or confounded by the process of neuroselection, whereby children select different active and sedentary behaviours according to their levels of cognition.^12^ It may be possible that children with higher levels of cognition select into specific types of physical activity and sedentary behaviour, some of which (eg, reading or doing homework) may be beneficial for other aspects of life, such as academic achievement and job success. We previously argued that self-selection of health behaviours, particularly physical activity, may be restricted during early childhood, given the limited levels of independence at this age.^13^ However, as children approach adolescence, increased independence may present new opportunities for children to engage in health behaviours.^14 15^ Consequently, cognition at this age may be important for the onset and formation of life-long health behaviours. This may be particularly important given that health behaviours tend to track from childhood into adulthood.^16–20^ Furthermore, persistent exposure to unhealthy behaviours across the life course is likely to have the greatest risk to health.^21 22^ These associations may also vary according to the type of cognitive function in question. Analysis of the 1970 British cohort found that verbal ability at age 10 was more strongly associated with engagement in physical activity and a healthy diet at age 30 than non-verbal ability,^6^ suggesting that verbal abilities may be more important for selection of these health behaviours. Still, very little is known about how verbal and non-verbal abilities in early childhood are associated with other unhealthy lifestyle choices, such as smoking and alcohol consumption. Poorer spatial ability has been associated with higher depressive symptoms in middle-aged men^23^ and a higher risk of falls in older adults; however, how this specific domain of cognition in childhood is associated with health behaviours has not been studied.^24^

Our primary aim was to establish whether neuroselection occurred during early childhood. Specifically, we investigate whether cognition relating to verbal, non-verbal and spatial ability at ages 3–7 were associated with (1) physical activity, (2) sedentary behaviour, (3) having ever tried a cigarette and (4) having ever tried alcohol at age 11.

## Methods

### Millennium Cohort Study

The Millennium Cohort Study (MCS) is a prospective study of a representative sample of British children born between September 2000 and January 2002. Children were identified from the records of Child Benefit, which covers nearly all families in the UK aside from a small group of children with non-national parents with recent or temporary immigration status.^25^ A stratified sampling approach was employed, oversampling individuals from minority ethnic groups and disadvantaged areas, to ensure adequate representation of such groups. Information was collected from 18 552 families on 18 818 children at 9 months of age from one parent (usually the child’s mother). Further surveys were administered at the ages of 3, 5, 7 and 11 years. Data were collected from 15 590, 15 246, 13 857 and 13 287 families when children were aged 3, 5, 7 and 11, respectively.

### Exposure: verbal, non-verbal and spatial ability

Cognitive functioning has been assessed in the MCS since the second wave of data collection when children were aged 3. Assessments have included a battery of tests from the British Ability Scales (BAS).^26^ All cognitive measurements were collected in the child’s home by trained interviewers. When the children were 3 years old, the tests included the BAS Naming Vocabulary scale, which is used to assess the spoken vocabulary of children. Spoken vocabulary is a key component for development of written language.^27^ This was accompanied at age 5 years with the BAS Picture Similarities and Pattern Construction scales, which are used to assess non-verbal reasoning skills and spatial problem solving, respectively. There was a correlation between naming vocabulary scores at ages 3 and 5 (r=0.6). When the children were 7 years old (sweep 4), the assessments included the Pattern Construction scale, as well as BAS Word Reading scale. There was also a correlation between pattern construction scores at ages 5 and 7 (r=0.6). The Word Reading scale is used to assess verbal ability and requires respondents to name single words. T scores or standardised BAS scores were generated using BAS normative data set for each test,^28^ with a higher score indicating better performance. A T score of 50 represents the same raw score as the mean of their norming group. A T score of 60 represents a score that is 1 SD above the mean of their norming group. Standardised scores for the Word Reading scale (mean±SD, 111±18) were transformed in the same manner; however, they do not have a mean of 50 and SD of 10. The BAS measures are nationally standardised, have shown good inter-rater reliability^29^ and have been shown to correlate strongly with measures of intelligence.^30^ Further information on the BAS measures is reported in detail elsewhere.^26^ We constructed a composite score for verbal and spatial abilities by calculating the mean of all standardised scores from the relevant tests. These scores were combined in order to derive a score for each domain of cognitive function. BAS Naming Vocabulary T scores at ages 3 and 5 and BAS Word Reading standardised score at age 7 were used to derive verbal ability. BAS pattern construction scores at ages 5 and 7 were used to derive spatial ability. BAS Picture Similarities T score at age 5 was used as a measure of non-verbal ability.

### Self-reported behaviours: physical activity, sedentary behaviour, smoking and alcohol consumption

At age 11, children were asked how often they engage in a number of health behaviours. They reported how often they played sports and active games outside of school, read for enjoyment outside of school, played games on the computer or games console outside of school and how often they exchanged messages with friends online. Response options for these questions were never, less often than once a month, at least once a month, at least once a week and most days. Children were also asked whether they had ever tried a cigarette (even a single puff) and whether they had ever consumed more than a few sips of an alcoholic drink. Response options for these questions were yes or no. Although these specific questions have not been validated, previous studies have demonstrated the reliability and validity of self-reported physical activity,^31^ tobacco^32 33^ and alcohol consumption^33 34^ in similar age groups. We also observed a weak but significant positive correlation between sports participation at age 11 and accelerometry derived time spent in moderate-to-vigorous physical activity (MVPA) at age 7 (rho=0.17) and a weak negative correlation between time spent reading at age 11 and time spent in MVPA at age 7 (rho=−0.10), which provides some evidence of convergent validity for the self-reported physical activity data.

### Covariates

A number of confounding factors were identified prior to analysis for inclusion in the models based on existing literature about socio-demographic, lifestyle and health factors in relation to cognition and physical activity.^35 36^ These included child’s age, maternal smoking status during pregnancy (smoker or non-smoker), income (above or in poverty), highest maternal qualification (GCSE grades D–G, GCSE grades A–C, A/AS levels, diploma in higher education, degree or above), ethnicity (white British or other) and strengths and difficulties score. The Strengths and Difficulties Questionnaire (SDQ) is a valid and reliable measure of child emotional and behavioural problems^37^ and was completed by parents when children were aged 7.

### Statistical analysis

Participants were categorised as having low (at least once a week or less) or regular (most days) participation in each physical activity and sedentary behaviour. Binary responses (yes/no) for having ever smoked and having ever consumed alcohol were used for analyses. Participants missing any of the BAS scores between ages 3 and 7 were excluded from analyses (n=8342). Descriptive statistics were reported for demographic, health behaviour and cognitive function variables. Differences in the proportion of children reporting regular participation in sport and active games according to engagement in each sedentary behaviour were examined using χ^2^ tests. Binary logistic regression was used to estimate the ORs of engaging in health behaviours at age 11 per 1 SD increase in verbal, non-verbal and spatial abilities in early childhood. Analyses adjusted for age, sex, maternal smoking status during pregnancy, income, maternal qualifications, ethnicity and strengths and difficulties score (model 1). Model 2 additionally included all cognitive measures at all ages in the models to determine whether the associations for each domain were independent of other cognitive domains. Variance inflation factors (VIFs) were estimated to evaluate multicollinearity between exposure variables. VIF values did not exceed 2, indicating no multicollinearity between exposure variables. We also tested for sex interactions and where significant models were run separately for boys and girls. Analyses were carried out using SPSS V.22 (IBM) and an alpha level P≤0.05 was used to determine significance.

## Results

A total of 8129 participants had complete data for self-reported health behaviours, covariates and BAS cognitive scores (at ages 3, 5 and 7). Descriptive characteristics for the analytic sample are presented in table 1. A total of 59.4% of the sample engaged in regular sport and active games. Proportions of engagement varied significantly according to engagement in sedentary behaviours. Compared with children with low levels of reading, fewer children who regularly read for enjoyment also regularly engaged in sport and active games (62.8% vs 55.5%, P<0.001). Similarly, compared with children with low levels of computer gaming, fewer children who regularly played computer games also regularly engaged in sport and active games (60.5% vs 58.4%, P=0.06). Conversely, compared with children with low levels of online messaging, a higher proportion of children who regularly messaged online also regularly engaged in sport and active games (57.3% vs 61.4%, P<0.001). Compared with those excluded from the analyses, the analytic sample was more likely to be female (50.9% vs 46.9%, P<0.001), white British (89.2% vs 77.2%, P<0.001), have higher educated mothers (≥A levels, 42.7% vs 26.1%, P<0.001), have mothers who did not smoke during pregnancy (25.4% vs 31.6%, P<0.001), have lower scores on the SDQ (7.0 vs 8.4, P<0.001), regularly read for enjoyment (46.7% vs 43.1%, P<0.001), have previously consumed alcohol (12.4% vs 10.5%, P<0.05), score higher on cognitive tests (eg, verbal ability composite score, 51.0 vs 47.3) and were less likely to be in poverty (27.6% vs 41.1%, P<0.001), regularly play computer games (53.8% vs 56.5%, P<0.05) and have ever smoked (2.3% vs 3.4%, P<0.001).

**Table 1:** Descriptive characteristics and early childhood cognitive function scores

Characteristic	% (n)	Mean±SD
Total sample	8129	
Age, mean±SD		10.7±0.5
Male	49.1 (3992)	
White British	89.2 (7255)	
Maternal highest qualification (≥A levels)	42.7 (3473)	
Poverty level income	27.6 (2243)	
Smoked during pregnancy	25.4 (2062)	
SDQ score		7.0±5.1
Sport and active games (% regularly)	59.4 (4827)	
Reading for enjoyment (% regularly)	46.7 (3798)	
Gaming on PC/games console (% regularly)	53.8 (4370)	
Online messaging (% regularly)	21.9 (1779)	
Ever smoked (% yes)	2.3 (184)	
Ever consumed alcohol (% yes)	12.4 (1007)	
Verbal ability composite score		51.0±10.0
Non-verbal ability composite score		56.4±10.0
Spatial ability composite score		52.9±8.8

PC, personal computer; SDQ, Strengths and Difficulties Questionnaire.

Table 2 shows the odds of participating in sport and active games and specific sedentary behaviours at age 11 according to verbal, non-verbal and spatial abilities from ages 3 to 7. A 1 SD increase in verbal ability was associated with reduced odds of participating in sport and active games at age 11 in boys and girls after adjusting for non-verbal and spatial abilities. Higher spatial ability was also associated with reduced odds of participating in sport and active games at age 11, but this was attenuated to non-significance in girls after adjusting for non-verbal and spatial abilities. Non-verbal ability was not associated with participation in sport and active games in boys or girls.

**Table 2:** Early life cognition and odds of regular engagement in sport and sedentary behaviours at age 11

	Model 1 OR (95% CI)		Model 2 OR (95% CI)	
	Boys (n=3992)	Girls (n=4137)	Boys (n=3992)	Girls (n=4137)
Sport and active games				
Verbal ability	0.87 (0.81 to 0.94)	0.80 (0.74 to 0.87)	0.91 (0.84 to 0.99)	0.81 (0.74 to 0.88)
Non-verbal ability	0.96 (0.90 to 1.03)	1.00 (0.94 to 1.07)	1.02 (0.95 to 1.10)	1.05 (0.99 to 1.13)
Spatial ability	0.86 (0.80 to 0.92)	0.92 (0.86 to 0.98)	0.88 (0.82 to 0.95)	0.96 (0.89 to 1.03)
Reading for enjoyment				
Verbal ability	1.52 (1.40 to 1.64)	1.46 (1.34 to 1.58)	1.47 (1.35 to 1.60)	1.48 (1.36 to 1.62)
Non-verbal ability	1.17 (1.10 to 1.25)	1.07 (1.00 to 1.14)	1.07 (1.00 to 1.15)	1.01 (0.95 to 1.08)
Spatial ability*	1.17 (1.10 to 1.26)	1.07 (0.99 to 1.14)	1.03 (0.95 to 1.11)	0.95 (0.88 to 1.03)
Gaming on PC/games console				
Verbal ability*	0.94 (0.87 to 1.02)	0.80 (0.74 to 0.87)	0.93 (0.86 to 1.02)	0.79 (0.72 to 0.86)
Non-verbal ability	0.99 (0.92 to 1.06)	0.97 (0.91 to 1.03)	0.99 (0.92 to 1.07)	1.00 (0.93 to 1.07)
Spatial ability	1.00 (0.93 to 1.07)	0.98 (0.91 to 1.05)	1.02 (0.95 to 1.10)	1.05 (0.97 to 1.13)
Online messaging				
Verbal ability*	1.00 (0.91 to 1.10)	1.05 (0.96 to 1.15)	1.07 (0.96 to 1.18)	1.06 (0.96 to 1.17)
Non-verbal ability*	0.88 (0.81 to 0.96)	1.01 (0.95 to 1.09)	0.89 (0.0.81 to 0.98)	1.02 (0.94 to 1.09)
Spatial ability*	0.90 (0.83 to 0.98)	0.98 (0.90 to 1.05)	0.92 (0.84 to 1.01)	0.95 (0.88 to 1.04)

ORs reflect the likelihood of regular participation in each activity per 1 SD increase in cognition score. For sport and active games, reading for enjoyment, gaming on PC/games console, this reflects participation on most days. For online messaging, this reflects participation at least once a month.

Model 1 included only one cognitive measure as an exposure per regression and adjusted for age, maternal smoking status during pregnancy, income, maternal qualifications, ethnicity and strengths and difficulties score.

Model 2 adjusted for variables in model 1 and included all cognitive scores at ages 3–11 as exposure measures.

*Significant sex interaction detected (P<0.05).

PC, personal computer.

Higher non-verbal ability was associated with increased odds of reading for enjoyment at age 11, but this was attenuated to non-significance in girls after adjusting for verbal and spatial abilities. The largest effect size was observed between verbal ability and reading for enjoyment at age 11 in boys (OR 1.48, 95% CI 1.36 to 1.62) and in girls (1.47, 95% CI 1.35 to 1.60). Spatial ability was not associated with reading for enjoyment in boys or girls.

Cognitive function was not associated with computer gaming at age 11 in boys, but higher verbal ability was associated with reduced odds of computer gaming in girls after adjustment for non-verbal and spatial abilities. Higher non-verbal (OR 0.88, 95% CI 0.81 to 0.96) and spatial abilities (OR 0.90, 95% CI 0.83 to 0.98) were associated with reduced odds of regular online messaging in boys at age 11. Non-verbal ability remained significantly associated after adjusting for other domains of cognitive function (OR 0.89, 95% CI 0.0.81 to 0.98). Verbal ability was not associated with online messaging in boys or girls.

When boys and girls were combined, higher verbal ability was associated with reduced odds of attempting smoking by age 11 (table 3). Higher non-verbal ability was associated with reduced odds of alcohol consumption by age 11, but verbal ability was marginally associated with increased odds of alcohol consumption.

**Table 3:** Early life cognition and odds of ever tying smoking and alcohol at age 11 (n=8129)

	Model 1 OR (95% CI)	Model 2 OR (95% CI)
Ever smoked		
Verbal ability	0.70 (0.59 to 0.84)	0.69 (0.57 to 0.84)
Non-verbal ability	1.03 (0.88 to 1.19)	1.14 (0.97 to 1.33)
Spatial ability	0.88 (0.76 to 1.02)	0.94 (0.80 to 1.12)
Ever consumed alcohol		
Verbal ability	1.06 (0.98 to 1.15)	1.09 (1.00 to 1.19)
Non-verbal ability	0.93 (0.87 to 1.00)	0.92 (0.85 to 0.99)
Spatial ability	1.00 (0.93 to 1.07)	1.00 (0.92 to 1.08)

ORs reflect the likelihood of having ever smoked and having ever consumed alcohol per 1 SD increase in cognition score.

Model 1 included only one cognitive measure as an exposure per regression and adjusted for age, sex, maternal smoking status during pregnancy, income, maternal qualifications, ethnicity and strengths and difficulties score.

Model 2 adjusted for variables in model 1 and included all cognitive scores at ages 3–11 as exposure measures.

## Discussion

In this sample of British children, early childhood cognition was associated with participation in health behaviours at age 11. Specifically, these findings suggest that children with higher cognition select into some healthier behaviours, including avoidance of smoking and alcohol consumption. However, they may also select into some adverse health behaviours, such as less sport and exercise and more time in some sedentary behaviours. Higher verbal ability was more strongly and consistently associated with healthier behaviours at age 11 than non-verbal and spatial abilities.

There may be a number of potential explanations for these observations, and future research should explore potential pathways. First, it may be possible that children with higher cognition have an increased preference for and ability to carry out specific sedentary tasks, such as reading, which may increase their participation in these activities and displace time spent in more active pursuits. For example, regular engagement in sport and active games was significantly lower in children reporting regularly reading for enjoyment. Motivation for reading is a key determinant for time spent reading in childhood,^38^ and cognition may drive this association. Another possible explanation may be that children with higher levels of cognitive function with lower athletic ability are more likely to make perceptions about their ability and consider avoiding this behaviour to avert the stigma and teasing by peers. Furthermore, it may be that the perceived adverse effects and threat of smoking and alcohol to health may be more profound than the consequences of an inactive lifestyle in children. Health information regarding smoking and alcohol may be easier for children to interpret and act on than physical activity advice. The results also highlight that there are gender differences in the importance of specific components of cognitive function for selection of health behaviours.

### Comparison with other studies

#### Cognitive function, physical activity and sedentary behaviour

A number of studies have shown that cognition in childhood is positively associated with more favourable lifestyle behaviours and health outcomes in adulthood,^1–7^ providing evidence for the neuroselection hypothesis.^3^ This is the first study that we are aware of to examine whether the neuroselection theory applies to physical activity and sedentary behaviours in childhood. In contrast with previous studies in adults, we found that higher cognition in early childhood was associated with lower odds of participation in sport and active games at age 11. We also found that higher early childhood cognition was associated with higher levels of reading in boys and girls at age 11, which may be beneficial for academic achievement and future job prospects.^39^ Conversely, higher non-verbal abilities were associated with lower levels of online messaging in boys at age 11 plausibly because boys with higher non-verbal abilities are less comfortable with written communication. Further, higher verbal ability was associated with reduced odds of computer gaming in girls. These results suggest that the influence of cognitive function in early childhood on the self-selection of sedentary behaviours is context specific. It may be possible that children with higher levels of cognition engage more in sedentary behaviours such as reading, which in turn displaces time in sport and other sedentary behaviours such as computer gaming. Reading ability is associated with a number of health outcomes,^40^ and other sedentary tasks such as homework and music are beneficial for cognitive development.^11 41^ Thus, interventions should focus on retaining these sedentary behaviours but displacing less cognitively engaging tasks, such as TV viewing and computer gaming, with physical activity. We previously showed in this cohort that the association between childhood sedentary behaviours and later cognitive function was also context specific, suggesting that some (eg, homework) but not all (eg, TV viewing) sedentary behaviours are neuroprotective.^11^ In the present study, we also demonstrated that specific domains of cognitive function in early childhood were more strongly associated with participation in health behaviours at age 11. Verbal ability was most strongly and consistently associated with physical activity and sedentary behaviours at age 11, which is consistent with previous research.^6^ Verbal abilities are important for reading comprehension^42^ and thus may be crucial for the interpretation of health information and management of health behaviours. Higher verbal ability may also increase preference for related sedentary tasks such as reading.

#### Cognitive function, smoking and alcohol consumption

Children who experiment with smoking and alcohol are more likely to partake in these behaviours in later life,^19 20^ and so understanding their determinants may help intervention strategies prevent early consolidation of such behaviours. Higher cognition in childhood has been shown to be associated with a reduced risk of smoking in adulthood.^4^ Similarly, there is evidence that childhood cognition is associated with reduced risk of binge drinking and heavy alcohol consumption in adulthood.^5 43^ However, there is little evidence whether the choice to take up smoking or drink alcohol in childhood is associated with early childhood cognition. Consistent with previous findings in adults, our results showed that early childhood verbal ability was associated with reduced odds of attempting cigarette smoking by age 11. Non-verbal ability was associated with reduced odds of drinking alcohol by age 11. These results support the neuroselection hypothesis by suggesting that higher early life cognition is associated with avoidance of harmful behaviours in childhood.

The major strengths of this study are its large representative sample and prospective design including various measures of cognition during early childhood. This allowed us to examine the associations of cognition during early childhood with health behaviours at age 11. It also allowed investigation into the influence of specific types of cognition on these behaviours. This study also has some limitations. First, some aspects of cognition such as memory may also be important for the selection of health behaviours, but these were not explored. Second, only self-reported physical activity and sedentary behaviour were available at age 11 in this cohort. While self-reported data allow us to interrogate context-specific associations, self-reported data may be subject to recall and social desirability bias.^44^ Moreover, the list of sedentary behaviours examined was not exhaustive. Early life cognition may also be associated with other sedentary behaviours such as television viewing, for example. Further studies should examine these associations with objective measures of activity such as accelerometers. Lastly, our final sample differed from those excluded from analyses according to a number of characteristics including ethnicity, socioeconomic status, exposure to unhealthy behaviours and cognitive ability, which may reduce the representativeness of our data.

In conclusion, the results suggest that higher cognition in early life is associated with avoidance of hazardous behaviours, such as smoking and alcohol consumption, but also avoidance of some healthy behaviours, such as sport and exercise. Physical activity interventions may need to target children with higher levels of cognition to increase participation, particularly those with high levels of verbal ability. Approaches to improve cognitive ability in early childhood may improve children’s ability to successfully manage their health behaviours on a wider scale.

**What is already known on this subject**

- Higher cognitive function in childhood is associated with a reduced risk of unhealthy lifestyle choices in adulthood.
- Individuals with higher cognitive function select into healthier behaviours, a theory known as neuroselection.

**What this study adds**

- Early life cognitive function at ages 3–7 years is associated with engagement in health behaviours at age 11.
- Children with better cognitive function may select into some healthier behaviours (eg, avoidance of smoking and alcohol consumption) by age 11.
- They may also select into some less healthy behaviours (eg, less sport and active games and more sedentary time.)
